# Real-world safety of ulinastatin: a post-marketing surveillance of 11,252 patients in China

**DOI:** 10.1186/s40360-022-00585-3

**Published:** 2022-07-16

**Authors:** Jin Li, Meijun Li, Liren Li, Lin Ma, Ailin Cao, Aiping Wen, Wenge Chen, Lingling Li, Yan Liang, Jianxiong Deng

**Affiliations:** 1 Pharmacy Department, Guangzhou United Family Hospital, Guangzhou, 510335 Guangdong China; 2 Guangdong Province Pharmacological Society, No. 753 East Dongfeng Road, Guangzhou, 510080 Guangdong China; 3 Department of Pharmacy, Nanfang Hospital, Southern Medical University, Guangzhou, 510515 Guangdong China; 4 Managed Service Organization, Guangdong Hospital of Traditional Chinese Medicine, Guangzhou, 510120 Guangdong China; 5 Department of Medicines and Devices, The First Affiliated Hospital of Naval Military Medical University (Shanghai Changhai Hospital), Shanghai, 200433 China; 6 Department of Pharmacy, Beijing Friendship Hospital, Capital Medical University, Beijing, 100050 China; 7 Department of Industrial Engineering, College of Mechanical and Electrical Engineering, Guangdong University of Technology, Guangzhou, 518049 Guangdong China

**Keywords:** Ulinastatin, Post-marketing reevaluation, Phase IV study, Rational drug use, Adverse drug reaction

## Abstract

**Background:**

The safety assessment of ulinastatin can guide clinical practice. The present study aimed to investigate the real-world safety of ulinastatin in China.

**Methods:**

This multicenter study retrospectively analyzed the post-marketing surveillance data of consecutive patients treated with ulinastatin between August 2014 and June 2017 in the general wards and the intensive care units (ICU) of nine hospitals in China. Adverse drug reactions/adverse drug events (ADRs/ADEs) were collected and evaluated in a post-marketing database.

**Results:**

A total of 11,252 consecutive patients were included in the study: 7009 ICU patients and 4243 general ward patients. Eleven patients with ADRs/ADEs were observed, including nine ICU patients and two general ward patients. The clinical manifestations were liver dysfunction (*n* = 5 ICU cases, *n* = 1 general case), thrombocytopenia (*n* = 2 ICU cases, *n* = 1 general case), leukopenia (*n* = 1 ICU case), and rash (*n* = 1 ICU case). During the study period, the drug ADR/ADE rate of ulinastatin injection was 0.98‰ (11/11,252 × 1000‰). Among the 11,252 valid patients, only 327 received ulinastatin in accordance with the drug specifications. After excluding unreasonable drug use, the calculated ADR rate was 3.06‰ (1/327 × 1000‰) (95% confidence interval: 0.0‰-17.1‰). In ICU and general ward patients, the use of other drugs combined with ulinastatin was associated with the occurrence of ADRs/ADEs (100% with ADRs/ADEs vs. 0% in controls, *P* < 0.001).

**Conclusions:**

The incidence of ADRs/ADEs of ulinastatin is < 5‰. The ADRs/ADEs involved limited organs, mainly the skin, gastrointestinal tract, and blood. In most cases, the ADRs/ADEs gradually alleviated or recovered after drug withdrawal. The inappropriate/off-label use of ulinastatin should be the focus of surveillance.

**Supplementary Information:**

The online version contains supplementary material available at 10.1186/s40360-022-00585-3.

## Background

Centralized hospital-based monitoring is one of the methods used to determine the safety and usage of drugs in the real world [1, 2]. Unbiased data can be obtained by monitoring multiple hospitals in specific areas [3]. Data monitoring also reflects the adverse drug reactions (ADRs) in real-world clinical practice [3]. Compared with the self-reported system, centralized hospital monitoring can summarize the rate of ADRs and risk factors of clinical use [3]. Centralized hospital-based monitoring is more suitable for collecting ADRs with a low incidence.

Ulinastatin was approved in 1999 by the China Food and Drug Administration (CFDA). It is a glycoprotein with protease inhibitor activity and has been used for decades in Asia for acute respiratory distress syndrome [4], pancreatitis [5], multiorgan failure [6], and sepsis [7]. Ulinastatin inhibits trypsin, hyaluronidase, α-chymotrypsin, and granulocyte elastase and prevents the release of lysosomal content [8–10]. Ulinastatin also has anti-inflammatory properties by reducing tumor necrosis factor (TNF)-α, interleukin (IL)-6, and IL-8 [11, 12].

Clinical trials showed that the adverse drug events (ADEs) of ulinastatin include dizziness, injection site pain, decreasing white blood cell (WBC), nausea, vomiting, allergic dermatitis, phlebitis, and rhinorrhea [13–18]. Still, some clinical trials did not report the safety of ulinastatin [12, 19] or analyze the adverse effects [4, 10]. In addition, the clinical trials usually select the cases and cannot represent the actual situation [8, 20, 21]. Hence, the post-marketing safety reevaluation of ulinastatin for injection would be helpful for the guidance of clinical use.

This multicenter study aimed to investigate the safety of ulinastatin and analyzed the data from a post-marketing database about the clinical use and safety of ulinastatin in China. A large-scale investigation on its safety might assist the policy formulation and implementation of the administration department and guide the rational use.

## Methods

### Study design and data source

This multicenter study retrospectively analyzed the post-marketing data of patients who received ulinastatin (Guangdong Techpool Bio-pharma Co., Ltd., Guangdong, China) between August 2014 and June 2017 in general wards and intensive care units (ICUs) of nine hospitals in China.

The study protocol was approved by the Ethics Committee of Guangdong Provincial Hospital of Chinese Medicine (No. B2014–056-01) as the lead center. The study was carried out in accordance with the relevant guidelines and regulations (“*Opinions on Reforming the Review and Approval System for Drugs and Medical Devices*” in 2015 by the CFDA and “*Regulations for the Implementation of the Drug Administration Law of the People’s Republic of China*” in 2016 by the State Council). The clinical data were from nine hospitals. The requirement for informed consent was waived by the Ethics Committee of Guangdong Provincial Hospital of Chinese Medicine.

### Routine assessments

The use of ulinastatin for injection, such as indications, routes, dosage, solvents, duration, concentration, and course, were evaluated as package inserts. According to the package insert, for acute pancreatitis and chronic recurrent pancreatitis, in the initial stage, 100,000 U are dissolved in 500 mL of 5% glucose injection or 0.9% sodium chloride injection for intravenous drip, administered 1–3 times/day, 1–2 h apart, and reducing the dose as the symptoms disappear. For acute circulatory failure, 100,000 U are dissolved in 500 mL of 5% glucose injection or 0.9% sodium chloride injection for intravenous drip pr in 5–10 mL of 0.9% sodium chloride injection slowly, 1–3 times/day, 1–2 h apart. The doses can be appropriately increased or decreased according to age and symptoms. In China, all off-label uses only need to be authorized by the director of the clinical department rather than by the pharmaceutical therapy and safety committee of the hospital.

The ATC coding was used for the drugs, and the ICD10 codes were used for the diseases. Only safety events related to the rational use of ulinastatin were ADRs according to the package inserts, and safety events related to the off-label use of ulinastatin were ADEs. The pharmacists investigated patients’ information, medication treatment, and ADRs and filled in the case report forms (CRFs). All ADEs were identified by the physicians and reviewed by experts to confirm their relevance to ulinastatin. ADRs/ADEs were assessed following the Common Adverse Event Evaluation Standard 4.0 (CTCAE 4.0).

### Data collection

The following data were collected: 1) information (sex, age, allergic history, department, diagnosis, etc.), 2) drug use (doses, route, frequency, solvent, volume, course, mixed with other drugs (in a bag or bottle), and concomitant drugs), 3) ADRs/ADEs including allergies [22, 23], facial swelling [24], itching, reddening, phlebitis [25], etc. according to the package inserts and previous studies, and 4) lab tests, vital signs, and radiological reports.

### Statistical analysis

All data were processed using SPSS 18 (SPSS Inc. Released 2009. PASW Statistics for Windows. Chicago: SPSS Inc. USA). The continuous data were presented as means ± standard deviation and evaluated using the independent samples *t*-test. Categorical data were presented as n (%) and evaluated using the chi-square test. Logistic regression was used to calculate the odds ratio (OR) and 95% confidence interval (CI) of the risk factors. Two-sided *P* < 0.05 was statistically significant.

The ADR/ADE cases were matched with non-ADR/ADE cases in a 1:4 ratio and according to clinical departments, diagnosis, sex, and age.

## Results

### Demographic data for the patients

Finally, 11,252 cases (mean age, 55.74 ± 16.20) received ulinastatin for injection, including 7009 (62.29%) ICU cases and 4243 (37.71%) general ward cases (Table 1).

**Table 1:** Characteristics of the patients

	Total (*n* = 11,252)	ICU (*n* = 7009)	General ward (*n* = 4243)
Age, years, median (range)	59.31 (46 days-105 years)	61.92 (46 days-104 years)	52.67 (11 months-105 years)
Age, year, mean ± SD	55.74 ± 16.20	58.37 ± 14.85	51.40 ± 17.35
Sex, n (%)			
Male	7323 (65.08)	4648 (66.31)	2675 (63.05)
Female	3929 (34.92)	2361 (33.69)	1568 (36.98)
Pregnancy, n (%)	48 (0.43)	12 (0.17)	36 (0.85)
Allergy history, n (%)	814 (7.23)	550 (7.85)	264 (6.22)
Hypertension, n (%)	2106 (18.72)	1562 (22.29)	544 (12.82)
Unstable angina, n (%)	1461 (12.98)	1443 (20.59)	18 (0.42)
Acute pancreatitis, n (%)	640 (5.69)	139 (1.98)	501 (11.81)
Consistency with the indications*, n (%)	1567 (13.93)	648 (9.25)	919 (21.66)
Mixed with other drugs	1739 (15.46)	1715 (24.47)	24 (0.57)
Combined with other drugs, n (%)	272 (3.88)	301 (7.09)	573 (5.09)

*, patients were treated with ulinastatin for diseases described in the package insert

Among the 7009 ICU cases, the youngest was 46 days old, and the oldest was 104 years old (mean age, 58.37 ± 14.85). There were 4648 males (66.31%) and 2361 females (33.69%, 12 pregnancies), and 550 (7.85%) with an allergy history. Hypertension was the most common comorbidity (*n* = 1562, 22.29%), followed by unstable angina pectoris (*n* = 1443, 20.59%). Among the 7009 cases, 648 (9.25%) met the indications, i.e., acute pancreatitis, acute exacerbation of chronic recurrent pancreatitis, and acute circulatory failure.

Among 4243 general ward cases, the youngest was 11 months old, and the oldest was 105 years old (mean 51.40 ± 17.35). There were 2675 males (63.05%) and 1568 females (36.95%, including 36 pregnancies); 264 (6.22%) cases had an allergy history. The main allergens were antibiotics, including penicillin, cephalosporins, and sulfonamides. The most common comorbidity was hypertension (*n* = 544, 12.82%), followed by acute pancreatitis (*n* = 501, 11.81%). The primary diagnoses were digestive diseases, circulatory diseases, and tumors. Among the 4243 cases, 919 (21.66%) met the indications.

### Ulinastatin usage and cumulative dose

During the study period, 7009 ICU cases were given ulinastatin 7933 times (Table 2). The course was 2 (1–138) days (means, 3.38 ± 5.20). The mean dose was 146.58 ± 271.30 million U, and the mean cumulative dose was 165.90 ± 307.98 million U. Among the 7009 cases, 4784 (60.31%) received ulinastatin mixed with sodium chloride injection according to package inserts, 1236 (15.58%) with 5% glucose injection, 1885 (23.76%) received off-label solvent (including invert sugar injection, fructose injection, 10% glucose injection, mixed sugar electrolyte injection, etc.), and 28 (0.35%) without solvent information. In addition, 2669 cases (33.64%) had a solvent volume of 20 ml, followed by 1848 cases (23.30%) with 250 ml and 1779 cases (22.43%) with 100 ml. The rational solvent dose was 500 ml in 11 cases, 5–10 ml in 692 cases as an intravenous infusion, and 5–10 ml in 31 cases as an intravenous pump (Supplementary Table S1).

**Table 2:** Usage of ulinastatin in clinical practice

	Total	ICU	General ward
Total use, times, n	12,719	7993	4726
Single dose consistent with the indications, times, n (%)	2878 (22.63)	991 (12.40)	1887 (39.93)
Single dose of 300 thousand U, times, n (%)	2751 (21.63)	1852 (23.17)	899 (19.02)
Single dose > 10,000 U, times, n (%)	9736 (76.55)	6938 (87.46)	2798 (59.19)
Single dose < 10,000 U, times, n (%)	45 (0.35)	4 (0.05)	41 (0.87)
Consecutive treatment time, day, median (range)	5 (1–141)	2 (1–138)	3 (1–141)
Usage times, day, mean ± SD (range)	3.83 ± 5.52 (1–196)	3.38 ± 5.20 (1–138)	4.59 ± 5.94 (1–196)
Average continuous single dose, million U, mean ± SD	145.70 ± 262.23	146.58 ± 271.30	144.22 ± 246.16
Average cumulative total dosage/patient, million U, mean ± SD	163.94 ± 302.96	165.90 ± 307.98	160.64 ± 294.27
Solvent use, times, n (%)			
Sodium chloride	8626 (67.82)	4784 (60.31)	3842 (81.29)
5% glucose	2066 (16.24)	1236 (15.58)	830 (17.56)
Others*	1928 (15.16)	1885 (23.76)	43 (0.91)
Unknown	39 (0.31)	28 (0.35)	11 (0.23)
Solvent volume, times, n (%)			
10 ml	2927 (23.01)	1447 (18.10)	1480 (31.32)
20 ml	3573 (28.09)	2669 (33.64)	904 (19.12)
100 ml	3102 (24.39)	1779 (22.43)	1323 (27.99)
250 ml	2397 (18.84)	1848 (23.30)	549 (11.62)
500 ml	720 (5.66)	250 (21.23)	470 (9.94)
Administration route, n (%)			
Intravenous drip	6480 (50.95)	4246 (53.52)	2234 (47.27)
Intravenous pump	2068 (16.26)	1913 (24.11)	155 (3.28)
Intravenous injection	4030 (31.68)	1774 (22.36)	2256 (47.74)
Prefilling	141 (1.11)	60 (0.75)	81 (1.71)
Dosing frequency, n (%)			
once daily	4837 (38.03)	2519 (31.75)	2318 (49.05)
twice daily	3523 (27.70)	1978 (24.93)	1545 (32.69)
3 time/day	2305 (18.12)	1562 (19.69)	743 (15.72)
4 time/day (off-label)	71 (0.55)	66 (0.83)	5 (0.11)
6 time/day (off-label)	7 (0.0055)	7 (0.09)	0 (0)
8 time/day (off-label)	4 (0.03)	1 (0.01)	3 (0.06)
Treatment just once, n (%)	404 (3.18)	386 (4.87)	18 (0.38)
Treatment immediately, n (%)	1507 (11.85)	1414 (17.82)	93 (1.97)
Mixed with other drugs, times, n (%)	2058 (16.18)	2015 (25.20)	43 (0.91)
Combined with other drugs, times, n (%)	12,719 (100.00)	7993 (100.00)	4726 (100.00)

*, others included: invert sugar injection, fructose injection, 10% glucose injection, and mixed sugar electrolyte injection

Finally, 4243 general ward cases received ulinastatin 4726 times. The median of a single course was 3 (1–141) days. The average cumulative course was 4.59 ± 5.94 (1–196) days, and 1 case lacked frequency information. The mean single dose was 144.22 ± 246.16 million U, and the mean dose was 160.64 ± 294.27 million U. Among the 4243 cases, 3842 (81.29%) received ulinastatin with sodium chloride injection as per drug instructions, 830 (17.56%) with 5% glucose injection, 43 (0.91%) used off-label solvents (including 10% glucose for injection, ringo, sodium lactate for injection, and other types of solvents), and 11 had missing solvent information. Regarding the solvent volume, 10 ml held a majority with 1480 (31.32%) cases, followed by 100 mL with 1323 (27.99%) cases, 20 ml with 904 (19.12%) cases, and 500 ml with 470 (9.94%) cases. Only 459 (9.71%) cases received rational solvent 500 mL during intravenous infusion, 1426 cases received 5–10 mL during intravenous infusion, and 35 cases received 5–10 ml during intravenous pump (Supplementary Table S1).

### Usage of single dosage and solvent

Among the 7933 ICU cases, 991 (12.49%) had single doses of 100,000 U, and the single doses were mainly 300,000 U (*n* = 1852, 23.17%) (Table 2). The single doses of 6938 (87.46%) cases exceeded the maximum of 100,000 U, and the doses of 4 cases (0.05%) were lower than the minimum 10,000 U recommended by the package inserts. An intravenous drip was the main route, with 4246 cases (53.52%), followed by an intravenous pump in 1913 cases (24.11%) and intravenous injection in 1774 cases (22.36%). Frequency was once daily in 2519 cases (31.75%), twice daily in 1978 (24.93%), three times daily in 1562 (19.69%), 1414 cases (17.82%) for stat use, and 386 cases (4.87%) for only once. Off-label frequency included 66 (0.83%) cases who received ulinastatin four times daily, 7 (0.09%) with six times daily, and 1 (0.01%) with eight times daily (Supplementary Table S1).

Among the 4726 general ward cases, single doses of 100,000 U were given to 1887 cases (39.93%). The doses in 2798 cases (59.19%) exceeded the maximum recommended, while the doses in 41 cases (0.87%) were lower than the minimum recommended. The intravenous injection was the main route, with 2256 cases (47.74%), 2234 (47.27%) as an intravenous infusion, 155 (3.28%) as an intravenous pump, and 81 (1.71%) as prefilling. Of the 4243 cases, the drug was administrated 4726 times, of which 2318 times with once daily (49.05%), 1545 (32.69%) with twice daily, 743 (15.72%) with three times daily, 93 (1.97%) with stat use, and 18 (0.38%) for only once. Off-label use frequency included 5 (0.11%) with four times daily and 3 (0.06%) with eight times daily (Supplementary Table S1).

### Drug combination

Combined drugs were used in 1715 ICU cases, with 2015 times. The mixed drugs mainly include troxerutin injection, sodium phosphocreatine for injection, and sodium monosialate tetrahexosaccharide ganglioside. There were 38 combined drugs. A total of 28 ICU cases were filled in the CRFs with 272 combined medication events. Cardiovascular drugs were the most used, with 55 (20.22%), followed by digestive and electrolytes and nutrition drugs with 43 and 39 (15.81 and 14.34%), respectively. The main drugs were 5% glucose, dopamine, and ambroxol injection.

In the general ward cases, other drugs were used in 24 cases, with 43 times. There were 14 mixed drugs, including insulin, magnesium isoxalate, and reduced glutathione. There were 243 cases with combined drug use, for 301 times. Among them, the electrolytes and nutritional drugs were the most common, with 64 cases (21.26%), followed by anesthetic and digestive drugs with 39 and 27 (12.95 and 8.97%), respectively. The main drugs were 0.9% sodium chloride injection, ambroxol, propofol, and omeprazole.

### Adverse drug reactions

In this study, 11 cases of ADR/ADE were observed, including nine ICU cases and two general cases. The clinical manifestations were abnormal liver function (3 ICU cases and 1 general case), liver function damage (1 ICU case), thrombocytosis (1 ICU case and 1 general case), thrombocytopenia (1 ICU case), leukocytosis (1 ICU case), rash (1 ICU case), and leukopenia (1 ICU case) (Table 3). Systems involved skin and accessory lesions, digestive system, and blood system. One case was evaluated as “probably relevant” and 10 as “possibly relevant”. The severity of ADRs/ADEs was graded grade 1–2 in 10 cases and grade 4 in 2 (Table 4). ADRs/ADEs occurred within 6 days after administration. After ADRs/ADEs, all cases stopped ulinastatin. Only one case of rash was treated with calamine lotion. All cases recovered or improved within 11 days after the occurrence of ADRs/ADEs without the reuse of ulinastatin again (Table 4).

**Table 3:** Safety of ulinastatin in clinical practice

Adverse drug reaction	ICU		General ward	
	patients (n)	events (n)	patients (n)	events (n)
**Possibly related**				
Abnormal liver function	3	3 *	1	1
Liver function damage	1	1	0	0
Thrombocytosis	1	1	1	1
Thrombocytopenia	1	1	0	0
Leukocytosis	1	1	0	0
Rash	1	1	0	0
**Probably related**				
Leukopenia	1	1	0	0
**Sum**	9	9	2	2

* 1 patient occurred grade 4 ADR/ADE of abnormal liver function, and the other 10 patients were all grade 1 or 2

**Table 4:** Occurrence and outcome of ulinastatin ADR/ADE

No.	ICU or General	Gender	Age	Events	Severity*	Treatment	Further treatment	Outcome	Duration of outcome	Relevance
1	General	Female	66	Liver function damage	1	None	None	Improved	5d	Possibly
2	General	Male	58	Thrombocytosis	1	Discontinuation	None	Recovered	11d	Possibly
3	ICU	Female	42	Abnormal liver function	1	Discontinuation	None	Recovered	4d	Possibly
4	ICU	Female	57	Thrombocytopenia	1	Discontinuation	None	Recovered	5d	Possibly
5	ICU	Male	36	Abnormal liver function	1	Discontinuation	None	Recovered	2d	Possibly
6	ICU	Male	53	Rash	1	None	Local use of calamine lotion	Recovered	8d	Possibly
7	ICU	Male	53	Thrombocytosis	4	Discontinuation	None	Improved	10d	Possibly
8	ICU	Male	57	Abnormal liver function	2	Discontinuation	None	Recovered	7d	**Probably**
9	ICU	Male	58	Leukocytosis	1	None	None	Recovered	1d	Possibly
10	ICU	Male	58	Leukopenia	1	Discontinuation	None	Improved	1d	Possibly
11	ICU	Male	76	Abnormal liver function	4	Discontinuation	None	Improved	1d	Possibly

* The severity grades of adverse events in the Common Adverse Event Evaluation Standard 4.0 (CTCAE) (1, 2, 3, 4)

The drugs used by the patients who experienced ADR/ADEs are shown in Supplementary Tables S1 and S2. Among the nine patients with ADR/ADEs in the ICU, a total of 100 combined drugs were used; the most common were electrolyte, acid-base balance, and nutritional drugs, followed by digestive system drugs, antimicrobial drugs, cardiovascular system drugs, and respiratory system drugs (Supplementary Table S2). Two general ward patients received 36 combined drugs. The most common were anesthetics, followed by cardiovascular system drugs, hematological system drugs, endocrine system drugs, and antimicrobial drugs (Supplementary Table S3).

### Univariable analyses after matching

In the ICU, 45 cases were analyzed in the univariable analyses after matching, including 9 cases with evaluated ADRs/ADEs (case group) and 36 without (control group). The case group was 54.58 ± 23.02 years old, and the control group was 55.13 ± 22.08 years old (*P* = 0.977). Only the combined drugs were statistically significant between the two groups (*P* = 0.001) (Table 5). A total of 100 combined drug use were observed in the case group, while the control group had no combined drug use. Electrolyte and nutritional drugs were the most common, with 21 cases, followed by digestive drugs and antibiotics, with 19 and 12 cases, respectively.

**Table 5:** Univariable analysis in the ICU patients after matching

	ICU		
	Case group (*n* = 9)	Control group (*n* = 36)	*P*
Age, years, mean ± SD	54.58 ± 23.02	55.13 ± 22.08	0.977
Sex, n (%)			> 0.999
Male	6	24	
Female	3	12	
Allergy history, n (%)	0	0	–
Disease history, n (%)	8	26	0.544
Infection history, n (%)	1	3	0.798
Surgery history, n (%)	5	11	0.311
Smoking/drinking/drug use, n (%)	1	4	> 0.999
Combined with other drugs, n (%)	9	0	**0.001**

Among the general ward cases, 10 cases were analyzed in the univariable analyses after matching, including two cases with ADRs/ADEs (case group) and eight cases without (control group). The age of the case group was 59.23 ± 2.66 years old, and the controls were 59.60 ± 2.30 years old. There were no obvious differences between the two groups regarding the history of infectious diseases and trauma surgery (all *P* > 0.05). Age, sex, food, and drug allergy history, and allergy history could not be evaluated. There was a difference (*P* = 0.002) between the two groups regarding combined drugs (Table 6). There were 36 combined drug uses in the case group, while the control group had not. Anesthesia drugs were used eight times, followed by cardiovascular and blood drugs (7 and 6 times, respectively).

**Table 6:** Univariable analysis in the general ward patients after matching

	General ward		
	Case group (*n* = 2)	Control group (*n* = 8)	*P*
Age, years, mean ± SD	59.23 ± 2.66	59.60 ± 2.30	–
Sex, n (%)			–
Male	2	8	
Female	0	0	
Infectious disease/trauma/surgery history, n (%)	0	4	0.124
Food and drug allergy history, n (%)	0	0	–
Allergic disease history, n (%)	0	0	–
Other disease, n (%)	1	4	1
Combined with other drugs, n (%)	2	0	**0.002**

### Adverse drug reaction rate

In the study, the ADE rate of ulinastatin injection was 0.98‰ (11/11,252 × 1000‰). However, the ADR cases group did not include unreasonable drug use. In the study, only 327 patients received ulinastatin according to package inserts, and excluding the cases of unreasonable drug use, the ADR rate of ulinastatin for injection was 3.06‰ (1/327 × 1000‰), and the 95% CI was 0.00‰-17.10‰.

## Discussion

Ulinastatin is generally well-tolerated and has few ADEs in clinical trials, but real-world evidence (RWE) of safety was lacking. Therefore, this multicenter post-marketing surveillance study aimed to investigate the real-world safety of ulinastatin in China. The RWE results suggest that the incidence of ulinastatin ADR/ADE is < 5‰. The ADRs/ADEs involve limited sites, mainly the skin, digestive system, and blood. In most cases, the ADRs/ADEs gradually alleviated or recovered after drug withdrawal. The inappropriate/off-label use of ulinastatin should be the important target of surveillance. RWE is important to the safety monitoring of drugs. Indeed, RWE studies examine the actual patients who receive the drug in opposition to clinical trials, in which highly selected patients are treated with the drug. In clinical trials, patients with comorbidities and confounding factors are usually excluded from determining the exact effects of the drug, but such patients will receive the drug in actual practice and might be more susceptible to developing ADRs/ADEs. RWE cannot replace clinical trials, but they complement each other.

During the RWE study, the ADR/ADE rate of ulinastatin injection was 0.98‰ (11/11,252 × 1000‰). Among the 11,252 valid cases collected in the study, only 327 received ulinastatin following the package inserts. Excluding unreasonable drug use, ADR/ADE rate was 3.06‰ (1/327 × 1000‰), which was “occasional” according to the ADR/ADE classification standard. It is lower than the seven ADR/ADE cases among 497 cases (1.41%) in the meta-analysis by Chen et al. [18]. However, the meta-analysis only included randomized controlled trials of ulinastatin vs. traditional Chinese medicine combined with ulinastatin.

The ADRs/ADEs in the RWE study involved skin and accessory damage, digestive, and blood system. 1 case of ADR/ADE was moderate, 1 case was life-threatening, and the other 8 cases were mild. All cases were alleviated or recovered 11 days after drugs’ were discontinued without intervention. Therefore, using ulinastatin is safe, and ADR/ADE is rare. The above RWE data help identify methods, characteristics and focuses of safety monitoring of drug usage.

As the rate of ADRs/ADEs might vary with the geographical distribution, population, living environment, and habits of the patients, it is necessary to conduct a nationwide investigation on a large scale. More than 10,000 cases were included in the study, but some very rare ADRs/ADEs might have been missed. Therefore, it is suggested that within the allowed limits of the human and financial resources, the total sample size should be expanded to > 100,000 cases. Data collection is still ongoing.

Most of the ADRs/ADEs occurred on the first day of medication, suggesting that the patients should be particularly closely observed on the first day of medication, especially during the first 30 min after infusion, and stay alert to the occurrence of ADRs/ADEs to be able to react promptly by stopping or reducing the dosage. Still, in this study, ADR/ADE occurred within 6 days after medication, suggesting that clinical attention should be paid during hospitalization since ADRs/ADEs can still be observed after ulinastatin administration.

In addition to the ADRs/ADEs, this study characterized the use of ulinastatin in nine hospitals in China. The results revealed that most of the cases (97.10%) did not receive ulinastatin according to the product monograph. The type and dose of solvent and the number of uses per day were off-label in most cases. It is supported by a previous retrospective study in China that showed that the dosage was inconsistent with the recommendations in many cases [26]. Nevertheless, even when including the off-label use of ulinastatin, the rate of ADR/ADE was still low. Still, surveillance should be performed by the pharmacy departments to ensure that the drug is used according to the recommendations. It constitutes the basis for the rationale and safe use of drugs in hospitals. The recommended solvents for ulinastatin are saline or glucose solutions, with a volume of 500 ml for infusion, 5–10 ml of solvent during intravenous injection, and 5–10 ml of solvent for an intravenous pump. The maximal dosing per day should not exceed three times, and the dose should be 100,000–200,000 U.

This study has some limitations. Despite its large sample size, the actual rate of ADRs/ADEs was low, probably preventing the observation of rare ADRs/ADEs. In addition, because of the low occurrence of ADRs/ADEs, the logistic regression results should be taken with caution. Secondly, about two-thirds of the patients were from the ICU and were with severe conditions that might have hidden some mild ADRs/ADEs, resulting in an underestimation of the ADR/ADE rate. The analyzable data was limited. Finally, ulinastatin is only approved in China, India, South Korea, and Japan, limiting the scope of the present study.

## Conclusions

In conclusion, the RWE ADR/ADE rate of ulinastatin is < 5‰. ADR/ADEs are observed in cases of ulinastatin combined with other drugs. The ADRs/ADEs included liver dysfunction, thrombocytopenia, leukopenia, and rash. In most cases, the ADR/ADEs gradually resolved after discontinuing the drug. This RWE study revealed inappropriate/off-label uses of ulinastatin for most patients. We should focus on monitoring and education during the use of ulinastatin.

## Supplementary Information

**Additional file 1.**

## Data Availability

The datasets used and analyzed during the current study are available from the corresponding author on reasonable request.

## Abbreviations

ICU: Intensive care unit
ADR/ADE: Adverse drug reaction/adverse drug events
CFDA: China Food and Drug Administration
WBC: White blood cell
CRF: Case report forms
OR: Odds ratio
CI: Confidence interval

## References

[1] Cowling BJ, Feng S, Finelli L, Steffens A, Fowlkes A (2016). Assessment of influenza vaccine effectiveness in a sentinel surveillance network 2010-13, United States. Vaccine.

[2] Thomas RE, Lorenzetti DL, Spragins W, Jackson D, Williamson T (2011). Active and passive surveillance of yellow fever vaccine 17D or 17DD-associated serious adverse events: systematic review. Vaccine..

[3] Zhao Y, Chen Z, Huang P, Zheng SW, Xu QL, Shi C (2019). Analysis of Ornidazole injection in clinical use at post-marketing stage by centralized hospital monitoring system. Curr Med Sci.

[4] Leng YX, Yang SG, Song YH, Zhu X, Yao GQ (2014). Ulinastatin for acute lung injury and acute respiratory distress syndrome: a systematic review and meta-analysis. World J Crit Care Med.

[5] Tsujino T, Komatsu Y, Isayama H, Hirano K, Sasahira N, Yamamoto N (2005). Ulinastatin for pancreatitis after endoscopic retrograde cholangiopancreatography: a randomized, controlled trial. Clin Gastroenterol Hepatol.

[6] Atal SS, Atal S (2016). Ulinastatin - a newer potential therapeutic option for multiple organ dysfunction syndrome. J Basic Clin Physiol Pharmacol.

[7] Linder A, Russell JA (2014). An exciting candidate therapy for sepsis: ulinastatin, a urinary protease inhibitor. Intensive Care Med.

[8] Ma PP, Zhu D, Liu BZ, Zhong L, Zhu XY, Wang H (2013). Neutrophil elastase inhibitor on proliferation and apoptosis of U937 cells. Zhonghua Xue Ye Xue Za Zhi.

[9] Yuan L, Zhu X (2014). The role of neutrophil elastase and its inhibitors in acute respiratory distress syndrome: an update. Zhonghua Wei Zhong Bing Ji Jiu Yi Xue.

[10] Zhang X, Zhu Z, Jiao W, Liu W, Liu F, Zhu X (2019). Ulinastatin treatment for acute respiratory distress syndrome in China: a meta-analysis of randomized controlled trials. BMC Pulm Med.

[11] Inoue K, Takano H, Shimada A, Yanagisawa R, Sakurai M, Yoshino S (2005). Urinary trypsin inhibitor protects against systemic inflammation induced by lipopolysaccharide. Mol Pharmacol.

[12] Xu CE, Zou CW, Zhang MY, Guo L (2013). Effects of high-dose ulinastatin on inflammatory response and pulmonary function in patients with type-a aortic dissection after cardiopulmonary bypass under deep hypothermic circulatory arrest. J Cardiothorac Vasc Anesth.

[13] Chen Q, Hu C, Liu Y, Liu Y, Wang W, Zheng H (2017). Safety and tolerability of high-dose ulinastatin after 2-hour intravenous infusion in adult healthy Chinese volunteers: a randomized, double-blind, placebo-controlled, ascending-dose study. PLoS One.

[14] Abraham P, Rodriques J, Moulick N, Dharap S, Chafekar N, Verma PK (2013). Efficacy and safety of intravenous ulinastatin versus placebo along with standard supportive care in subjects with mild or severe acute pancreatitis. J Assoc Physicians India.

[15] Jing L, Mao YS (2013). Clinical assessment of Chinese traditional medicine preparation in the treatment of severe sepsis. Chin Hosp Pharm J.

[16] Zhao GK, Liu YH (2013). Clinical research of Xuebijing combined with ulinastatin on severe sepsis. Lab Med Clin.

[17] Chen B, Wan XF, Wang JH (2017). Application observation of Xuebijing injection combined with ulinastatin in patients with severe pneumonia complicated with sepsis. JETCM..

[18] Chen G, Gao Y, Jiang Y, Yang F, Li S, Tan D (2018). Efficacy and safety of Xuebijing injection combined with Ulinastatin as adjunctive therapy on Sepsis: a systematic review and Meta-analysis. Front Pharmacol.

[19] Sun R, Li Y, Chen W, Zhang F, Li T (2015). Total ginsenosides synergize with ulinastatin against septic acute lung injury and acute respiratory distress syndrome. Int J Clin Exper Pathol.

[20] Ramagopalan SV, Simpson A, Sammon C (2020). Can real-world data really replace randomised clinical trials?. BMC Med.

[21] Kim HS, Lee S, Kim JH (2018). Real-world evidence versus randomized controlled trial: clinical research based on electronic medical records. J Korean Med Sci.

[22] Weilan W, Yan S (2009). One case had a reaction caused by intravenous drip of Ulinastatin. China Pharmaceuticals.

[23] Jun C (2020). A case of anaphylactic shock caused by Ulinastatin. China Modern Appl Pharm.

[24] Jie M, Sixi Z, Yanqing S (2016). A case of delayed allergic reaction and facial swelling caused by ulinastatin for injection. Pract Med Clin.

[25] Xiao SH, Luo L, Liu XH, Zhou YM, Liu HM, Huang ZF (2018). Curative efficacy and safety of traditional Chinese medicine xuebijing injections combined with ulinastatin for treating sepsis in the Chinese population: a meta-analysis. Medicine (Baltimore).

[26] Zhu J, Liu Q, Cheng G, Zhang Z, Wang X (2020). A retrospective study of the effectiveness of ulinastatin in the treatment of sepsis. J Emer Crit Care Med.

