# Supplementary material for: Real-world safety of ulinastatin: a post-marketing surveillance of 11,252 patients in China
Source: BMC Pharmacol Toxicol. 2022 Jul 16;23:51. doi: 10.1186/s40360-022-00585-3 (PMC9288682; doi:10.1186/s40360-022-00585-3)
Supplement: Supplementary file 1 — Additional file 1. [file 40360_2022_585_MOESM1_ESM.docx]

**Supplementary material**

**Participating hospitals**

The data were from Guangdong Hospital of Traditional Chinese Medicine, Beijing Anzhen Hospital, Beijing Friendship Hospital Affiliated to Capital Medical University, Changhai Hospital Affiliated to Naval Medical University, First Affiliated Hospital of Xi’an Jiaotong University, First Affiliated Hospital of Guangzhou Medical University, Nanfang Hospital of Southern Medical University, Dongguan Kanghua Hospital, and Foshan Hospital of Traditional Chinese Medicine hospital.

**Supplementary Table S1.** Analysis of the irrational use of ulinastatin for injection

| Types of irrational use | Details | Total cases | | Cases of ADE | |
| --- | --- | --- | --- | --- | --- |
|  |  | ICU (n=7,009) | General  (n=4,243) | ICU | General |
| Irrational dose | Single dose above recommended | 6,162 | 2,630 | 6 | 2 |
| Irrational choice of solvent | The solvent was not 5% glucose injection or 0.9% sodium chloride injection. | 32 | 32 | 0 | 0 |
|  | The solvent was not sodium chloride injection required in the instruction during intravenous injection. | 21 | 58 | 0 | 0 |
| Irrational solvent dose | Too small solvent volume for intravenous drip injection | 179 | 762 | 0 | 0 |
|  | Too large solvent volume for intravenous injection | 219 | 293 | 0 | 0 |
|  | Too large solvent volume for intravenous pump injection | 5 | 4 | 0 | 0 |
| Unreasonable frequency of dose | Beyond recommended | 1 | 0 | 0 | 0 |
| Extended duration of drug use | Continuous use over 20 days | 6 | 4 | 0 | 0 |
| Off-label drug use |  | 235 | 282 | 2 | 0 |
|  | Total cases without rational drug use: | 10,925 | | 10 | |
|  | Total trial cases: | 11,252 | | 11 | |
|  | Total cases with rational drug use: | 327 | | 1 | |

Note: A single case is recorded only once for multiple unreasonable cases.

**Supplementary Table S2.** Cases of combined drugs of patients occurring ADR/ADE in ICUs.

| Type of combined drugs | Cases (n) | % |
| --- | --- | --- |
| Electrolyte, acid-base balance, and nutritional drugs | 21 | 21.00 |
| Digestive system drugs | 19 | 19.00 |
| Antimicrobial drugs | 12 | 12.00 |
| Cardiovascular system drugs | 11 | 11.00 |
| Respiratory system drugs | 10 | 10.00 |
| Immune system drugs | 6 | 6.00 |
| Nervous system drugs | 6 | 6.00 |
| Chinese patent medicine | 6 | 6.00 |
| Anesthetics | 3 | 3.00 |
| Urinary system drugs | 2 | 2.00 |
| Endocrine system drugs | 2 | 2.00 |
| Hematological system drugs | 1 | 1.00 |
| Drugs for mental disorders | 1 | 1.00 |
| Total | 100 | 100.00 |

**Supplementary Table S3.** Cases of combined drugs of patients occurring ADR/ADE in general wards.

| Type of combined drugs | Case (n) | % |
| --- | --- | --- |
| Anesthetics | 8 | 22.22 |
| Cardiovascular System Drugs | 7 | 19.44 |
| Hematological system drugs | 6 | 16.67 |
| Endocrine system drugs | 3 | 8.33 |
| Antimicrobial drugs | 3 | 8.33 |
| Respiratory system drugs | 2 | 5.56 |
| Electrolyte, acid-base balance, and nutritional drugs | 2 | 5.56 |
| Antitumor drugs | 1 | 2.78 |
| Drugs for mental disorders | 1 | 2.78 |
| Digestive system drugs | 1 | 2.78 |
| Nervous system drugs | 1 | 2.78 |
| Urinary system drugs | 1 | 2.78 |
| Total | 36 | 100 |
